# Latent profile analysis for quality of life in older patients

**DOI:** 10.1186/s12877-022-03518-1

**Published:** 2022-11-11

**Authors:** Lidia Băjenaru, Alexandru Balog, Ciprian Dobre, Rozeta Drăghici, Gabriel-Ioan Prada

**Affiliations:** 1 University Politehnica of Bucharest, Bucharest, Romania; 2 National Institute for Research and Development in Informatics, Bucharest, Romania; 3 Doctoral School of Economic Informatics, Bucharest University of Economics Studies, Bucharest, Romania; 4 National Institute of Gerontology and Geriatrics “Ana Aslan”, Bucharest, Romania; 5 University of Medicine and Pharmacy “Carol Davila”, Bucharest, Romania

**Keywords:** Quality of life, WHOQOL-BREF instrument, Older patient, Latent profile analysis

## Abstract

**Background:**

Quality of life (QOL) is a complex concept known for being influenced by socio-demographic characteristics, individual needs, perceptions and expectations. The study investigates influences of such heterogeneous variables and aims to identify and describe subgroups of older patients who share similar response patterns for the four domains (physical health, psychological health, social relationships and environment) of World Health Organization Quality of Life instrument, Short Form (WHOQOL-BREF).

**Methods:**

The sample used included older Romanian patients (N = 60; equal numbers of men and women; mean age was 71.95, SD = 5.98). Latent Profile Analysis (LPA) was conducted to explore quality of life profiles with the four WHOQOL-BREF domains as input variables. Differences between profiles were analysed by MANOVA and ANOVAs as a follow-up.

**Results:**

The LPA results showed that the three-profile model was the most suitable and supported the existence of three distinct QOL profiles: low and very low (28.3%), moderate (63.3%) and high (8.4%). The relative entropy value was high (0.86), results pointed to a good profile solution and the three profiles differed significantly from one another.

**Conclusion:**

Our results reveal heterogeneity within the older adult sample and provide meaningful information to better tailor QOL improvement programs to the needs of older patient groups, especially those designed for patients of profiles related to poorer QOL in different domains.

## Background

Quality of Life (QOL) is a complex concept, approached in various disciplines that is interpreted and defined in several ways. Assessment of the quality of life is an important goal in medical and health research and involves a variety of target groups and research models. Many instruments have been developed for QOL assessment [1–3].

Among the countless tools developed for QOL assessment, WHOQOL-BREF is one of the best-known generic questionnaires, covering four domains: Physical health, Psychological health, Social relationships and Environmental health [4, 5].

The WHOQOL-BREF has been evaluated and applied in various countries, in different contexts and to general or specific populations [6–10]. At international level, numerous initiatives have been taken on ageing that support the importance of quality of life of older people in particular. There are a limited number of studies in scientific publications using WHOQOL-BREF to measure the QOL among older people [11–15]. There are no published WHOQOL-BREF studies on samples of Romanian participants therefore there are no comparable databases for data analysis.

Most studies in the field of QOL have tested the psychometric properties of the questionnaire and used variable-centred approaches (e.g., regression analysis, confirmatory factor analysis). These analyses are based on the relationships between variables and consider the studied samples as homogeneous, without considering the possibility that these relationships may differ in subgroups of participants [16].

An alternative approach is the person-centred approach that pays attention to the heterogeneity of individual response patterns and defines unique subgroups in a sample. This is suitable for assigning people into homogeneous subgroups and comparing quantitatively and qualitatively different subgroups (“profiles” or “classes”). There are different data analysis strategies commonly used in person-centred research, such as: Latent Class Analysis (LCA), Latent Profile Analysis (LPA) and Latent Transition Analysis (LTA) [17]. These techniques have become popular and have been applied in psychology, education, management, marketing, medical and health research.

Several studies have used person-centred approaches and different indicators to identify health profiles of older people for various purposes [18–20]. In these studies, QOL profiles have not been directly identified by using QOL variables. QOL was discussed in terms of classes or profiles, and researchers examined the associations between QOL and identified classes.

A few studies have identified QOL classes using responses to QOL questionnaires grouping variables in the general populations, community adults and students. For instance, De Maeyer et al. [21] and Buitenweg et al. [22] used the Lancashire Quality of Life Profile (LQOLP) scale in a sample of opiate-dependent individuals and a sample of older persons with severe mental health problems, respectively. In each of these studies, the LCA identified three classes of QOL. Other studies used the 5-domain EuroQol questionnaire (EQ-5D) to examine the heterogeneity in lung cancer population [23] and in a sample of older adults [24]. The LCA identified three classes and four classes, respectively. In another QOL profile study, Kelly et al. [25] used EUROHIS-QOL-8-item index in a population of people seeking treatment for substance dependence. The latent classes identified through LCA were: low, moderate, and high QOL.

In a person-centred approach, the focus is on relationships among variables related to participants to identify subgroups of individuals based on their response patterns to a set of variables. In the case of the older people, there is heterogeneity in their health and quality of life [26]. Due to the fact that elderly patients have different socio-demographic characteristics, needs and perceptions regarding their health status, their profiles are not necessarily equal [27]. The person-centred approach can identify the unobserved heterogeneity in the older population and generate categories of older people.

The aim of this study is to identify subgroups of older patients in our target group, 60 participants from the National Institute of Gerontology and Geriatrics “Ana Aslan” Romania, who share similar response patterns to the four domains of WHOQOL-BREF. Latent Profile Analysis (LPA) has been applied to study the heterogeneity of QOL among older people, as a data analysis commonly used in a person-centred approach. To our knowledge, there have been no studies that have used LPA on the WHOQOL-BREF to investigate quality of life in older individuals.

The use of LPA methods for small samples is specified in the literature. In these cases simpler models (fewer indicators and classes) and “well separated” classes may be appropriate. Potential analysis problems with small sample sizes include poorly performing fit indices, convergence failures, and failure to discover classes with low membership. Also, it is mentioned that for simple LCA models with a pair of well-separated classes, a sample size of up to 30 may be sufficient [28].

### Methods

#### Participants

The study was conducted in the European project vINCI [29]. The vINCI project developed a non-invasive monitoring application for the older people, using sets of extensible technologies, smart devices, in order to detect early symptoms of deficiencies associated with old age [29–31]. The design of the vINCI application is based on the patient profile model which is the input to provide personalised healthcare. The patient profile is used as evidence to assess the impact of the vINCI solution on perceived quality of life, allowing for appropriate adjustment of caregiver support [30].

The target participants in this study were older individuals, the patients 65 years of age and older admitted to the National Institute of Gerontology and Geriatrics (NIGG) “Ana Aslan” Bucharest on the geriatric ward, from January to July 2021, were considered for inclusion in the study and then evaluated against exclusion.

The inclusion criteria were: age ≥ 65 years, signed Informed Consent, preserved basic functional independence, adequate compliance with study protocol.

The exclusion criteria included: any acute medical condition; any surgery in the last three months; major neurocognitive disorder; moderate and severe depression; existing disability (needs human help in one or more basic activities of daily living); angina pectoris; uncontrolled high blood pressure; heart arrhythmias that could interfere with functionality; any terminal illness; frailty syndrome; risk of falls; any condition that might limit mobility (e.g. Parkinson disease, severe arthritis, stroke sequel); visual severe impairment. Exclusion criteria were documented by patients’ records including medical examination, medical charts and medical history.

The study included a total of 60 participants. The older adults completed the WHOQOL-BREF, the questionnaire for monitoring quality of life specified in the proposal of vINCI project, using the smart tablet. All participants signed the informed consent and data was collected after the study protocol was approved by the Ethics Committee of NIGG. The study design and measurement protocol are described elsewhere [29, 32].

#### Measures

The QOL questionnaire was the Romanian version of the WHOQOL-BREF. There was a legal agreement with the World Health Organisation (WHO), which granted a licence to use the Licensed Materials in accordance with their terms and conditions. WHOQOL-BREF includes 26 items (questions), 24 of these items are divided in one of four domains: physical health (seven items), psychological health (six items), social relationships (three items), and environmental health (eight items). Two items assess the perception of overall quality of life and general health [4, 5]. All items are rated on a 5-point scale, with higher scores indicating higher QOL.

The domain scores are the sum total score for each question within the domain, and finally, all the scores were transformed in the range 0-100 according to WHOQOL-BREF guidelines [4]. We used the cut-off point of 60 points to formulate the feedback and recommendations for the older patients, according to other similar studies [32, 33]. A total score of at least 60 points identified patients with a good QOL and a score below 60 points pointed to poor QOL. Socio-demographic variables collected in the study included gender, age, education level, marital status and health status self-reported by answering the first part of the WHOQOL-BREF questionnaire.

Person-centred analysis can be a useful approach for studying the quality of life of older people. Using LPA we can separate older patients into subgroups based on their self-assessment on WHOQOL-BREF domains which refer to quality of life. The identification of groups with distinct QOL profiles may be beneficial to clinicians, as profile-based results can be interpreted with reference to categories of patients and QOL data can be applied individually.

### Data analysis

Latent Profile Analysis (LPA) was conducted to investigate the optimal number of latent profiles that describe the patients’ perceptions for each of QOL domains. Models with one to four profiles were tested. All analyses were conducted in Mplus 7.4 using robust maximum likelihood estimation [34]. Based on the guidelines for fit indices in the literature [28, 35, 36], we used informational criteria (AIC, BIC and SABIC) in which lower values indicated superior fit and likelihood-based tests (VLMR-LRT and BLRT) which compared the fit between a *k*-profile solution with a *k*-1-profile solution. A non-significant value (p ≥ .05) for a *k*-profile solution supported the *k*-1 profile solution.

To evaluate the classification accuracy, the relative entropy was reported, values ranging from 0.0 to 1.0 with higher values indicating greater accuracy. We examined the average posterior probability of profile membership. Values ≥ 0.80 indicated a good profile solution.

Each solution was evaluated for its theoretical meaningfulness. To support the interpretation of the chosen solution, *z*-scores with a mean 0 and standard deviation 1 were calculated and used. Differences between profiles were analysed by multivariate analysis of variance (MANOVA) and ANOVAs as a follow-up.

## Results

### Descriptive statistics

The sample consisted of 60 participants with a mean age of 71.9 (SD = ± 5.98; range of 65–85) years. The sample included equal numbers of men and women. The majority had primary and secondary education (73.3%) and more than half (61.7%) of the participants were married (Table 1).

**Table 1:** Sample characteristics (N = 60)

Gender, *n* (%)			
	Male		30 (50.0)
	Female		30 (50.0)
Age			
	Mean (SD)		71.95 (5.98)
	Range, *n* (%)		
	65–70 years		31 (51.7)
	71–85 years		29 (48.3)
Marital status, *n* (%)			
	Married		37 (61.7)
	Single/ Separated/ Widowed		23 (38.3)
Education, *n* (%)			
	Primary/ Secondary school		44 (73.3)
	Tertiary/ Higher education		16 (26.7)
Health status, *n* (%)			
	Healthy		17 (28.3)
	Unhealthy		43 (71.7)

The descriptive statistics and correlations of the grouping variables used are presented in Table 2. The mean values were similar for four grouping variables (Physical - PHY, Psychological - PSY, Social - SOC, Environmental - ENV), with environment domain (ENV) having the highest mean. Regarding correlations between the grouping variables, we found that there were positive and high correlation between PHY and PSY, between PSY and SOC, and moderate correlations in other cases.

**Table 2:** Descriptive statistics and correlations

Variables	M	SD	PHY	PSY	SOC
PHY	64.40	18.90	1		
PSY	75.28	12.19	0.66^**^	1	
SOC	72.64	11.60	0.39^**^	0.50**	1
ENV	78.59	10.84	0.43^**^	0.45**	0.37**

M, Mean; SD, Standard Deviation; PHY, Physical; PSY, Psychological; SOC, Social

** Correlation considered significant at the 0.01 level (2-tailed)

#### Latent profile analysis (LPA)

LPA was performed with the four domains as input variables (Physical - PHY, Psychological - PSY, Social - SOC, Environmental - ENV). Models with one to four profiles were tested. Model selection was based on examination of the currently most recommended statistical model fit criteria. The optimum model selected included the solution with three-latent profiles.

**Fig. 1:**
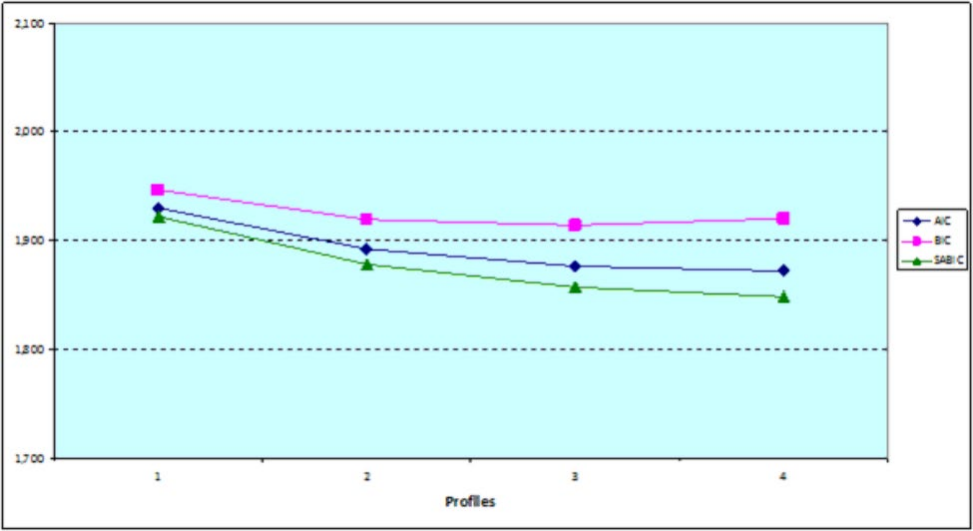
Comparison of the information criteria

As shown in Table 3, the AIC and SABIC information criteria did not suggest a specific solution. AIC and SABIC continued to improve (decreased) for each alternative model. Indeed, the BIC information criterion suggested that the optimum solution is the model with three latent profiles. The graphical representation “elbow plot” (Fig. 1) showed that the BIC continued to improve (decreased) up to three profiles model, and then deteriorated (increased) in the four profiles model.

**Table 3:** Comparison of fit indices between models

	AIC	BIC	SABIC	*Entropy*	*VLMR-LRT* *p-value*	*BLRT* *p-value*
1	1,929.92	1,946.67	1,921.51	1.000	-	-
2	1,891.75	1,918.98	1,878.09	0.774	0.023	0.00
**3**	**1,876.10**	**1,913.79**	**1,857.18**	**0.864**	**0.075**	**0.00**
4	1,872.04	1,920.21	1,847.87	0.915	0.250	0.14

The relative entropy value was high (0.86), and dropped within the recommended value (> 0.80). The mean posterior probabilities that respondents belonged to the latent profile to which they were assigned, were very high (0.91, 0.96, and 0.94).

The VLMR-LRT values remained significant (*p* < .05) up to two-profile model and then became non-significant (Table 3). These values indicated a two-profile solution. The BLRT value remained significant (*p* < .05) up to three-profile model and then became non-significant for the four-profile model (*p* = .14). Adding a fourth profile produced no significant improvement. BLRT indicated that the optimum solution was with three latent profiles. As suggested in the literature [36, 37] the best indices to be considered were BLRT and BIC. Taking into account results obtained, we chose the solution with three profiles.

Therefore, the profiles were distinct. In each of the three profiles there were enough respondents (17, 38, and 5, respectively) and the percentage of respondents in each profile was more than 5% (28.3%, 63.3%, and 8.4%, respectively).

Assigning the name for latent profiles was based on a pattern of probability response to items of each latent profile and comparison of average values of grouping variables for each latent profile.

**Fig. 2:**
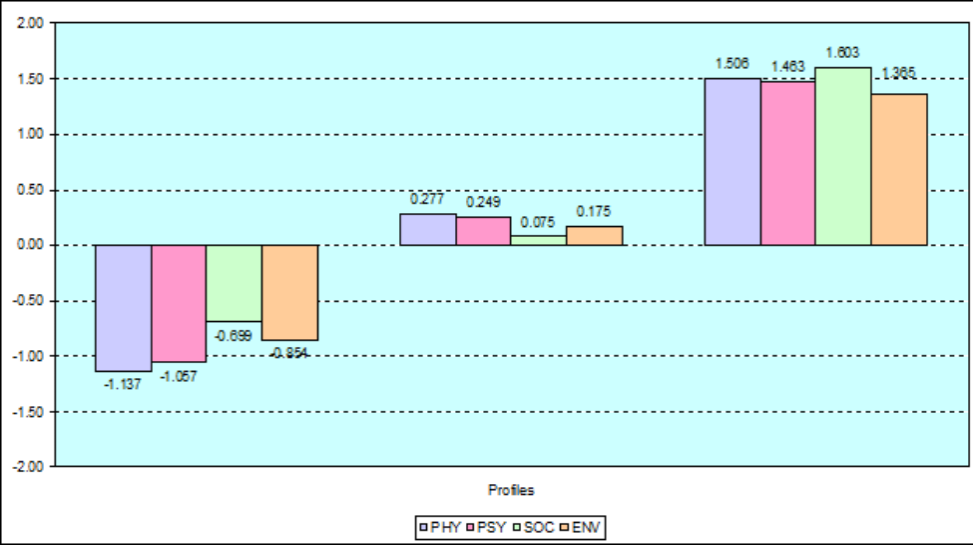
Graphs of profiles based on WHOQOL-BREF four domains. Note: The results were standardised (z-scores) to help interpret the histogram.

Results from the three-profile solution withheld were shown in Fig. 2. With this solution, profiles appeared differentiated in terms of levels.

The first profile consisted of 28,3% (17 patients) and was characterised by low and very low values for all four domains. All values were lower or much lower than the mean of the whole sample for each of the domains considered. Patients reported a very low degree of QOL in the Physical (PHY) and Psychological (PSY) domains. Also, patients reported a low degree of QOL in the Social (SOC) and Environment (ENV) domains. On the whole, members of this profile had perceptions about their low/ very low QOL. We called this profile “Low and very low QOL”.

The second profile was the largest and described 63.3% (38 patients) of the sample. It was characterised by values close to the mean values for all four domains. Overall, members of this profile had perceptions about moderate QOL for each of the domains considered. We called this profile “Moderate QOL”.

The third profile was the smallest, corresponding to 8.4% (5 patients) of the sample, and included patients with a very high level of QOL. On the whole, members of this profile had perceptions about their high QOL. We called this profile “High QOL”.

MANOVA results showed significant differences among the three profiles regarding all QOL domains (Wilks`s Lambda = 0.168, F (8,108) = 19.47, p < .001; partial eta squared η2 = 0.591). The multivariate effect size was large, namely 59.1% of the variance explained by the profile membership. The ANOVAs were all significant: F(2,57) = 47.59, p < .001, η2 = 0.625; F(2,57) = 33.66, p < .001, η2 = 0.542; F(2,57) = 19.17, p < .001, η2 = 0.402; F(2,57) = 20.71, p < .001, η2 = 0.421. The final profile solution accounted for 62.5%, 54.2%, 40.2%, and 42.1% of the variance in each domain. The three profiles differed significantly from one another.

## Discussion

For this Romanian sample, included older adults from the National Institute of Gerontology and Geriatrics “Ana Aslan”, Bucharest, findings of the study support the existence of three distinct QOL profiles: low and very low, moderate, and high QOL.

The largest group was characterised by moderate scores across all four QOL domains. This moderate QOL profile included an equal number of men and women, aged between 65 and 70 years, married, with secondary education and health problems.

The second largest group was the low and very low QOL profile having the lowest scores across all domains. Individuals of this profile had major problems with physical pain, were dependent on medication, with low energy and mobility, and also had problems with daily activities. They had negative feelings and problems regarding concentrating and the meaningfulness of life. Patients of this profile had problems with social relationships and perceptions about their poor environment. This group included women, aged between 71 and 85 years, married, with secondary education and serious health problems.

Individuals of the smallest group reported a very high degree of QOL for each of the domains considered. This high QOL profile included men, aged between 65 and 70 years, married, with higher education and health.

In the current study, findings indicated that the low and very low QOL profile groups had a significantly higher proportion of females than males. This suggests a potential need to tailor interventions according to gender differences.

The present study has been an important contribution. Findings reveal heterogeneity within older people samples suggesting necessity for developing quality of life improvement plans tailored for various groups of older individuals. Also, to our knowledge, the current study has been one of the first attempts to apply a person-centred approach (i.e., LPA) to understand QOL among older individuals and to explore the variability in QOL profiles among older patients.

There are several advantages to this approach. First, it can identify specific combinations of QOL domains that are optimal or suboptimal among older patients. Second, it analyses, describes the nature and prevalence of profiles (groups) and provides profile membership information for targeted interventions, especially those designed for patients of profiles related to poorer QOL in different domains. Describing QOL profiles of older patients provides helpful information concerning care that satisfies patients’ needs. Finally, research findings regarding QOL profiles can be interpreted with reference to groups of patients (who are grouped based on their similar responses/ attributes). Hence, identification of groups with distinct QOL profiles may be useful for integration of QOL data with data of clinical individual cases.

### Limitations

However, there are a few limitations to this research. First, our sample was too small and we had no means to increase our sample size, in order to assess whether and how QOL profiles differ across socio-demographic characteristics. We suggest future studies with larger samples to apply a person-centred approach. Second, while this study has been conducted with methodological rigour, the findings should be interpreted with caution. Our LPA lacked distal analyses not having means to conduct them, there for our results were limited. Third, the study looked at older patients in a single country (Romania). Replications are needed to further verify the QOL profiles in Romania and to assess whether similar groupings apply to other countries.

The findings cannot be generalised to the entire Romanian older population and even less to older people in other countries.

Despite limitations, findings of this study have several theoretical and practical implications. A person-centred approach (i.e., LPA) generates typologies of patients and uncovers unobserved heterogeneity of the older population.

## Conclusion

This study has been a notable contribution to awareness on QOL among older patients. Findings of our study support the existence of three distinct profiles of QOL among older patients and provide a better understanding of the heterogeneity of QOL. A profile-based perspective is a more intuitive way for patients/ caregivers/ doctors to understand older patients` quality of life.

## Data Availability

The dataset used and analysed during the current study is available from the corresponding author on reasonable request.

## Abbreviations

AIC: Akaike information criteria
ANOVA: analysis of variance
BIC: Bayesian information criteria
BLRT: Bootstrapped likelihood ratio test
ENV: environment domain
EQ-5D: EuroQol five dimensions questionnaire
LCA: Latent Class Analysis
LPA: Latent Profile Analysis
MANOVA: multivariate analysis of variance
NIGG: National Institute of Gerontology and Geriatrics
PHY: physical domain
PSY: psychological domain
QOL: quality of life
SD: standard deviation
SOC: social domain
SABIC: sample size adjusted Bayesian information criteria
WHO: World Health Organization VLMR-LRT:Vuong-Lo-Mendel-Rubin adjusted likelihood ratio test
WHOQOL-BREF: World Health Organization Quality of Life questionnaire, Short Form.
